# Biological Feedbacks as Cause and Demise of Neoproterozoic Icehouse: Astrobiological Prospects for Faster Evolution and Importance of Cold Conditions

**DOI:** 10.1371/journal.pone.0000214

**Published:** 2007-02-14

**Authors:** Pekka Janhunen, Hermanni Kaartokallio, Ilona Oksanen, Kirsi Lehto, Harry Lehto

**Affiliations:** 1 Department of Physical Sciences, University of Helsinki, Helsinki, Finland; 2 Finnish Meteorological Institute, Space Research, Helsinki, Finland; 3 Finnish Institute of Marine Research, Helsinki, Finland; 4 Department of Applied Chemistry and Microbiology, University of Helsinki, Helsinki, Finland; 5 Department of Botany, University of Stockholm, Stockholm, Sweden; 6 Nordic Institute for Theoretical Physics (NORDITA), Copenhagen, Denmark; 7 Department of Biology, University of Turku, Turku, Finland; 8 Tuorla Observatory, University of Turku, Turku, Finland; Max Planck Institute for Evolutionary Anthropology, Germany

## Abstract

Several severe glaciations occurred during the Neoproterozoic eon, and especially near its end in the Cryogenian period (630–850 Ma). While the glacial periods themselves were probably related to the continental positions being appropriate for glaciation, the general coldness of the Neoproterozoic and Cryogenian as a whole lacks specific explanation. The Cryogenian was immediately followed by the Ediacaran biota and Cambrian Metazoan, thus understanding the climate-biosphere interactions around the Cryogenian period is central to understanding the development of complex multicellular life in general. Here we present a feedback mechanism between growth of eukaryotic algal phytoplankton and climate which explains how the Earth system gradually entered the Cryogenian icehouse from the warm Mesoproterozoic greenhouse. The more abrupt termination of the Cryogenian is explained by the increase in gaseous carbon release caused by the more complex planktonic and benthic foodwebs and enhanced by a diversification of metazoan zooplankton and benthic animals. The increased ecosystem complexity caused a decrease in organic carbon burial rate, breaking the algal-climatic feedback loop of the earlier Neoproterozoic eon. Prior to the Neoproterozoic eon, eukaryotic evolution took place in a slow timescale regulated by interior cooling of the Earth and solar brightening. Evolution could have proceeded faster had these geophysical processes been faster. Thus, complex life could theoretically also be found around stars that are more massive than the Sun and have main sequence life shorter than 10 Ga. We also suggest that snow and glaciers are, in a statistical sense, important markers for conditions that may possibly promote the development of complex life on extrasolar planets.

## Introduction

According to geological evidence [1], Earth has experienced four main glacial periods: the Paleoproterozoic (2.1–2.5 Ga), the Neoproterozoic (600–950 Ma), the Carboniferous-Permian (260–360 Ma) and the ongoing Neogene (0–30 Ma). The Proterozoic (especially the Neoproterozoic) ice ages were the most severe in the sense that there is evidence for glaciation also at low paleolatitudes. Among the larger variations in climate are the Sturtian and Marinoan glaciations [2] which may have been nearly global in extent (sea ice at or near equator [3], [4]). At other times in its history, the planet has generally been too warm for significant continental glaciers to exist even in polar regions.

The Sun brightens about 1% per 100 Myrs when aging: from the Paleoproterozoic to the Neoproterozoic ice age the luminosity has increased from 0.83 L to 0.94 L [5]. The absence of glaciers during the Archean and Proterozoic Earth can be explained by a robust greenhouse which was probably CH_4_-dominated during the Archean [6] and CO_2_ and/or CH_4_ dominated during the Proterozoic [7]. The atmospheric CO_2_ is part of the carbon cycle where CO_2_ is exhaled from volcanoes, reacts with silicates to form carbonates, and ends up on the seafloor, from where it gets subducted by plate tectonics into the mantle and later re-emerges through volcanoes. The rate of silicate weathering increases with increasing surface temperature, which provides a stabilising thermostat mechanism [8]. Over timescales of >0.5 Myrs the CO_2_ outgassing rate (which depends on the speed of mantle convection and tectonic plates) must match the weathering rate. Thus not only Sun's brightness but also the vigour of mantle convection determines the surface temperature over >0.5 Myrs timescale. In the presence of a biosphere, burial of organic carbon also contributes to atmospheric carbon sequestration in addition to silicate weathering. The biosphere also effectively circulates carbon between its organic and inorganic states and through this process has been able to completely change the composition of the atmosphere. The early biosphere may have produced the methane greenhouse during the Archean and early Proterozoic and later, through oxygenic photosynthesis, has depleted the methane and regulated the ratios of atmospheric CO_2_ and O_2_, thus having a significant impact on the greenhouse effect of the atmosphere [9].

Thus the cooling of the Earth interior has slowed down mantle convection which has contributed to surface cooling due to its influence on the greenhouse effect. The brightening Sun has had an opposite effect to increase the effective temperature by 7 K from the Paleoproterozoic to the Neoproterozoic. The combined secular trend of the mean surface temperature is hard to predict and need not be large. However, both mechanisms led to the reduction of the importance of the greenhouse effect. This increased spatial and temporal temperature gradients, with corresponding increases in wind speeds and ocean currents. The climate has transformed from an early rather featureless and homogeneous greenhouse-dominated system to one containing important temperature gradients and thus exhibiting latitudinal and seasonal effects and a rich set of nonlinearities and feedbacks, among them the albedo feedback. This development would have also taken place without life, but biological effects have substantially modified it. Next we will discuss the feedback interactions involving the geophysical and biological factors.

**Figure 1:**
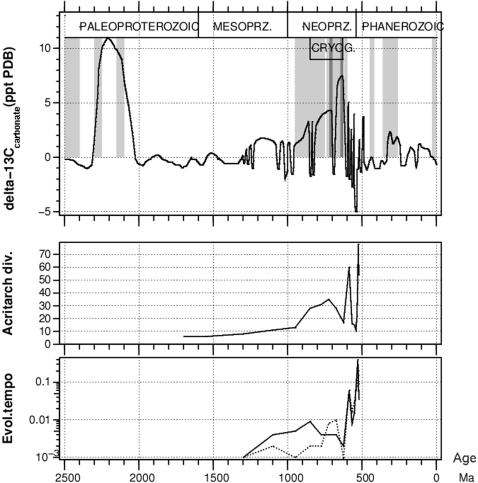
(a) Line: Deviation of δ^13^C/^12^C ratio from its standard Peedee belemnite (PDB) value in marine carbonate sediments. Adapted from [10]. Shadings show glacial periods: light shading means any geologic evidence of glaciation and darker shading means severe (global?) ice age. (b) Acritarch diversity; most acritarchs likely represent unicellular phytoplankton groups. (c) The evolutionary rate of acritarchs. Number of first apperance (solid) and last appearance (dotted) species per Myrs per species. Source for b and c is [30].

Figure 1a shows ^13^C/^12^C isotope fractionation in inorganic sediments [10] together with known glacial periods. Carbon fractionation, i.e. a relative enrichment of the heavier ^13^C in inorganic sediments, is caused by the preferential use of the lighter ^12^C isotope by the photosynthesis process, which leads to ^12^C enrichment of buried organic material and corresponding ^12^C depletion of inorganic sediments [10]. Hence, inorganic sediments enriched in ^13^C are indicative of high organic carbon burial rate. During the Phanerozoic eon one must be careful, however, because the carbonate-shelled algae link the organic and inorganic burial fluxes [10]; also during the Neoproterozoic the variations are so rapid that steady state was probably not reached [11]. The organic carbon burial rate was high during the early Paleoproterozoic, broadly simultaneously with the intensive glaciations. It has been suggested [12] that the Paleoproterozoic glaciations (at least the most recent one) were related to the radiation of cyanobacteria [13], [14] and to the high organic carbon burial rate due to their CO_2_ fixation.

At 1.3–2 Ga the isotope record shows only small variations, indicating that the relative rate of organic carbon burial and silicate weathering remained constant throughout this period. The small variations that one sees are likely due to changing continental positions having an effect on the efficiency of marine photosynthesis, e.g. by altering the nutrient supply, and thus on the organic carbon burial rate. At 1.3 Ga, organic carbon burial starts growing and the trend continues up to Ediacaran at 0.65 Ga. Again, this trend is modulated by shorter term variations with timescales consistent with plate tectonics. The modulations grow stronger with time because of decreasing greenhouse stabilisation and later by the albedo feedback associated with glaciations.

Figures 1 b and c show, respectively, the protist diversity and their evolutionary rate. The protist (unicellular eukaryote) biodiversity grows uninterruptedly until 0.7 Ma, with the evolutionary rate accelerating by factor of ten from 1.3 Ga to 0.8 Ga. The eukaryote diversity increases hand in hand with the organic carbon burial rate tracked by the isotope record. The ice-containing climate system is increasingly sensitive to changes in wind and ocean current patterns so that modest changes in continental positions sometimes cause large variations in the climate [15], [16]. These can be seen as a modulation in the net primary productivity and carbon burial rate, but without generating net losses of biodiversity until 0.7 Ga.

We interpret these data as follows. The driver of the biosphere-climate evolution process is the secular decrease in the relative importance of the greenhouse effect in determining the climate, which is due to the brightening Sun and the slowing mantle convection. This makes the marine ecological environment more diverse by increasing oceanic temperature contrasts and currents. The biosphere responds to these more varied conditions by developing more diverse eukaryotic algae which are mainly responsible for the increased primary productivity and organic carbon burial rate (eukaryotic algal cells are larger and thus faster-sinking than prokaryotic cyanobacteria and thus more easily generate buried carbon [17]). Eukaryotic algae existed already 2.1 Ga [18], [19] and today they dominate oceanic net primary production [20]. During the Neoproterozoic, however, there were no (widespread) animals to graze on the algae. Therefore, the modest increase in biodiversity stays in sync with the slow secular changes of the environment: in the absence of predation and macroscopic animals, interactions between species are still largely limited to short food chains dominated by (increasing) primary production and slow prokaryotic degradation of the organic matter.

The growing temperature contrasts eventually give rise to polar glaciers and sea ice (at 0.95 Ga, Figure 1a). The cold polar water masses initiate deep water formation and ocean ventilation, which increases the supply of nutrients to the photic zone [21]. Algal growth and organic carbon burial increase, more CO_2_ gets sequestered and the climate cools down. This establishes an algal-climatic positive feedback (net biological cooling), which causes the sea ice edge to expand towards the equator. The positive ice-albedo feedback contributes in the same direction. The details of the temporal variation are, however, strongly affected by changing continental positions through their effect on ocean currents.

The algal-climatic feedback saturates when tropical seas are cold enough to avoid thermal stratification under the prevailing wind conditions. At this stage, the planet is quite cold by modern standard and hosts strong algal growth. The sea ice edge is somewhere at mid-latitudes and continental glaciation is present. Stratigraphic data [22] indicates that active marine photosynthesis indeed continued during a Neoproterozoic glaciation. Part of this productivity may have been produced by ice-dwelling algae and cyanobacteria [23], [24].

The Cryogenian climate, with its general coldness and large swings (three major ice ages: Sturtian 720 Ma, Marinoan 635 Ma and Gaskiers 580 Ma) generated by the albedo feedback, would have continued indefinitely and only eventually been warmed by the slow solar brightening, unless something broke the algal-climatic feedback loop. We hypothesise that the rise of animals [25] was the necessary and sufficient agent for this breakage. Towards the end of the Neoproterozoic, the phytoplankton fossils contain increasing amounts of armor and ornamentation [17], which can be interpreted as defense against the grazing pressure from mesozooplankton. The establishment of new trophic levels in the marine food webs decreases the amount of buried organic carbon, because part of it is consumed in respiration before it ends up at the ocean floor. The production of above mentioned phytoplankton grazing defense systems also consumes part of the chemically bound energy which would otherwise be available for biomass production. In the benthos, burrowing animals consume organic carbon and by bioturbation increase the depth of oxygenated sediment layer, allowing bacteria to decompose organic matter more effectively, thus strongly enhancing the biological carbon cycling. The reduced organic carbon burial rate increases atmospheric CO_2_, warms the climate, reintroduces stratified oligotrophic seas in the tropic, which in turn further reduces algal primary productivity.

The radiation of animals was likely due to the high atmospheric oxygen levels [26], which was produced as a “side effect” of the algal-climatic feedback loop (amplified by the fact that cold water can contain more dissolved oxygen than warm water [21]). An indirect hint of high oxygen levels is that some unicellular organisms apparently grew to anomalously large size (over 1 mm) during the Neoproterozoic [27]. During and after the Cambrian, the oxygen concentration was possibly maintained high by a stabilising feedback between animals and O_2_: decreasing oxygen would reduce the competitiveness of zooplankton grazers, which would increase algal growth, organic carbon burial rate and the O_2_ abundance.

In our model, the rate of eukaryotic evolution proceeds at three speeds. First, the speed is very slow up to mid-Mesoproterozoic as it follows the timescale of the brightening of the Sun and the cooling of the planetary interior. Next, from the late Mesoproterozoic to the early Neoproterozoic, the rate of evolution is faster because of the nonlinear feedback between the climate and the biosphere, but interactions between species are still unimportant. Finally, the radiation of heterotrophic Metazoans starts an explosive increase of diversity which slows down only towards the end of Cambrian when animals have filled the readily available ecological niches.

At its peak, the evolution rate (Figure 1c) is more than two orders of magnitude higher than during the middle Mesoproterozoic. The total time taken by the whole process is dictated by the slowest rate in the first and longest interval. Since the evolutionary rate correlates with the rate of the climate change, determined both by the geophysical and biological processes, speeding up the geophysical processes would logically reduce the time needed to arrive from, say, cyanobacterial photosynthesis to Metazoans. In a planetary system which develops faster than ours, life could thus also develop faster. Therefore we should not neglect planets orbiting stars which are somewhat more massive the Sun when searching for evidence of complex life: the 4.2 Ga that it took on Earth to get to the Cambrian explosion need not be a universal timescale.

## Discussion

Generally speaking, at each stage the Earth system can be described by *N* important positive or negative feedback mechanisms, where by ‘important’ we mean the number of processes that must be included in order to accurately reproduce the dynamics. The number of relevant processes *N* can be small or large and it may vary from epoch to epoch. Sometimes one describes the large-*N* limit by the Gaia concept [28]. We think that ‘Gaia’ may be a reasonable approach for describing the Phanerozoic world, but the Proterozoic era is perhaps better described by a small-*N* model as we have tried to do in this paper.

Central to our argument concerning the entry into the Neoproterozoic icehouse is increased algal growth enabled by nutrients whose flow to the photic zone is possible in the absence of oceanic thermal stratification. Because cold water is heavy, the deep ocean always settles to roughly the same temperature as polar seas. The only way to remove thermal stratification globally is therefore to have the tropical surface waters at roughly the polar sea temperature. Clearly this is possible in two realms: (1) either the greenhouse effect is so strong that the surface temperature at pole and equator is essentially the same, or (2) the open waters are everywhere near freezing point. In both cases the oceans have no temperature gradients so that thorough vertical mixing may take place. Case 1 may never have materialised on Earth (except possibly during the methane greenhouse of the Archean), but we are suggesting that the Neoproterozoic world is an example of case 2.

Nutrients are brought to the ocean not only by vertical mixing, but also by rivers. If vertical mixing is efficient the river input may not be too important, but during the Paleo- and Mesoproterozoic when thermal stratification probably existed, its role may have been larger.

The role of suitable continental positions in creating the Cryogenian ice ages has often been stressed [3] and can be thought of as an alternative, non-biological hypothesis for the Cryogenian coldness. We think, however, that since the general coldness of the Neoproterozoic lasted for a long time (about 350 Myrs), the continental positions must have changed significantly during this long period, being sometimes favourable, sometimes unfavourable for glaciation. We agree that the *timings* of the individual glaciations (Sturtian, Marinoan and Gaskiers) of the Neoproterozoic were probably defined by continental positions (and possibly contributed by other factors such as changes in the cosmic environment [29]), but the *general coldness* of the Neoproterozoic is more likely due to biological reasons. In other words, we think that the start and end of the three major glaciations Sturtian (720 Ma), Marinoan (635 Ma) and Gaskiers (580 Ma) was defined by continental positions, but the fact that a *fourth* severe glaciation in the chain never realised itself was due to animals. The repetition interval between the Sturtian, Marinoan and Gaskiers glaciations was 55–85 Myrs, so if we assume that a similar trend was to continue, the fourth glaciation was expected 495–525 Ma, i.e. after the Cambrian explosion 530–542 Ma. Thus, in the light of these numbers it seems plausible and likely that a diverse Metazoan fauna with high ecological significance was already in place when a fourth severe glaciation was scheduled to hit due to evolving continental positions.

If the last Neoproterozoic ice age (Gaskiers) was timed by continental positions as we discussed above, the fact that nothing like it never repeated itself afterwards is remarkable. Something must have changed in the Earth system permanently at this point, and it cannot be the solar brightening alone which is too slow to reasonably account for it. Methane cannot have played a significant long-term role as a greenhouse gas any more at this stage, since from the continued existence of Metazoa we know that oxygen remained high throughout Phanerozoic and methane lifetime in a strongly oxygenated atmosphere is short. Thus regarding greenhouse gases other than water, only CO_2_ remains as an alternative, so something must have changed the carbon cycle. As we pointed out earlier, the biosphere can strongly affect the atmospheric composition. The temporal correlation of the increase of eukaryotic diversity and evolutionary tempo (Fig. 1 b, c) with the Neoproterozoic icehouse further supports the idea that these two processes were causally related.

Temperatures near freezing point are potentially important for multicellular life in at least two ways which are due to universal physical properties of water: (1) Cold water may contain more dissolved gases, including oxygen, than warm water [21]. This helps primitive multicellular heterotrophic organisms with larger body size and simple diffusive gas exchange to compete successfully with their unicellular peers. Afterwards the multicellulars developed an array of mechanisms such as blood circulation which enabled them to increase their body size and complexity further and to survive also in less oxygenated environments such as warm water. (2) To have the high dissolved oxygen in (cold) water, one of course needs also a high level of atmospheric O_2_. To reach that, high level of photosynthesis combined with reasonably large organic carbon burial fraction is needed. Among the best ways to achieve at least the photosynthesis part is to have nutrient-rich surface waters as widely distributed as possible and especially in the tropic where the photon flux is high. As discussed above, about the only way to reach that is to have the oceans everywhere cold and therefore isothermal, i.e. near the freezing point. Although this yields the high latitude seas to being ice-covered and therefore poorly producing and also the low temperature slows down life's chemical reactions, these are probably of lesser importance globally, because in spherical geometry the largest surface area is at low latitudes where the insolation is also optimal.

### Conclusions

We have brought up arguments to support the view that the generally cool climate during the Neoproterozoic which enabled the Sturtian, Marinoan and Gaskiers severe glaciations was due to reduced CO_2_ concentration caused by an enhanced organic carbon burial rate. The organic carbon was produced by algae thriving in cold oceans where thermal stratification was not hindering their nutrient supply. A byproduct of the enhanced carbon burial rate was an increase in atmospheric O_2_ which enabled multicellular animals to become competitive. The grazing pressure of the animals forced the primary producers to develop protective measures which reduced their ability to produce organic carbon. The burrowing action of benthic animals also helped to return organic carbon more efficiently into the atmosphere. Both effects tended to decrease the rate of organic carbon burial, whence the CO_2_ rose to a level which prevented a fourth severe glaciation after the Gaskiers from taking place during the Cambrian.

An outcome of the study is that one should not neglect stars which are somewhat more massive and more short-lived than the Sun when considering the possibilities for complex extraterrestrial life, since the rate of evolution during much of the Proterozoic was probably tied to solar brightening and cooling of the planetary interior. Another result is that when observing extrasolar terrestrial planets in the future, one should pay attention to finding signals of snow and ice for estimating statistically the commonality of those physical conditions which have the potential to provoke the development of complex life out of a possibly pre-existing microbial background.
